# Reconstruction of Surgical Defects From Early Carcinoma of Tongue Supported by Cross-Linked Hyaluronic Acid

**DOI:** 10.1097/SCS.0000000000012815

**Published:** 2026-05-05

**Authors:** Roberta Iaria, Paolo Vescovi, Carmen Mortellaro, Alberta Greco Lucchina, Anton Friedmann, Ilaria Giovannacci

**Affiliations:** * Department of Medicine and Surgery, Oral Medicine and Oral Surgery Laser Unit, University Center of Dentistry, University of Parma, Parma; † UniCamillus, Saint Camillus International University of Health Sciences, Rome, Italy; ‡ Witten-Herdecke University, Witten, Germany

**Keywords:** Hyaluronic acid, laser surgery, Nd:YAG laser, oral cancer, surgical reconstruction technique

## Abstract

**Background:**

Although treatment strategies for oral cancer of the tongue are determined based on the stage of the malignant lesion, surgical resection remains the cornerstone of therapy. Depending on the extent of the tumor, tissue resection can result in aesthetic and functional alterations of varying severity, with a significant impact on the patient’s quality of life.

**Methods:**

The following paper describes an innovative surgical technique performed under local anesthesia in an outpatient setting that combines the application of a cross-linked hyaluronic acid gel with a biocompatible membrane for the reconstruction of surgical defects after the excision of early-stage tongue carcinoma (T1–2, N0, DOI <5 mm). Five clinical cases of patients undergoing partial glossectomy with Neodymium-Doped Yttrium Aluminum Garnet (Nd:YAG) laser surgery are reported. Postoperative outcomes were evaluated at 1 week, 3 weeks, 2 months, and 1 year, focusing on wound healing, patient-reported symptoms, and functional recovery.

**Results:**

The evaluations showed that the proposed surgical technique was well tolerated by patients and resulted in favorable healing within 2 months. Furthermore, no complications or disease recurrence were observed.

**Conclusions:**

These results indicate that the proposed approach, performed under local anesthesia, may represent a valid reconstructive surgical option for early-stage tongue cancer, as it is associated with operative advantages such as the absence of a donor site and postoperative complications related to harvesting.

Oral cancer, together with pharyngeal carcinoma, is the sixth most common cancer in the world,^1^,^2^ with an estimated incidence of ∼389.846 new cases per year^1^,^3^,^4^ and a mortality rate of 188.438 (1.9%) worldwide.^4^,^5^ More specifically, the 5-year overall survival (OS) and disease-specific survival (DSS) are estimated to be 64.4% and 79.3%, respectively.^6^

Oral squamous cell carcinoma (OSCC) accounts for 90% of all oral malignancies and is the principal form of oral cancer.

To date, several agents and environmental factors are considered risk factors for oral cancer onset, and many others are currently under investigation, as their roles in pathogenesis are not yet entirely clarified (eg, gene polymorphisms).^7^

However, current literature reports a substantial increase in the incidence of oral cancer not related to specific risk factors. The literature of recent years has highlighted an upward trend in the percentage of young,^8^ nonsmoking, and nondrinking patients developing OSCC,^9^ with an annual rise of 0.7% of male patients and 1.7% of female subjects (SEER, Surveillance, Epidemiology and End Results data from 1973–2012).^9^,^10^ In 2020, the Global Cancer Observatory stated that ∼17% of OCSCC were diagnosed in young-adult patients.^5^,^11^

Among all the possible locations of oral cancer, the tongue is the most commonly affected site: it is known as the most common OSCC subsite.^12^

Compared with other forms of oral cancer, the Oral Tongue Squamous Cell Carcinoma (OTSCC) has a stronger propensity for local invasion and lymphatic spread, negatively impacting prognosis and making patient treatment more complicated.^13^,^14^ Indeed, among OCSCC patients, those with oral tongue cancer are more likely to experience 5-year cause-specific mortality (CSM) than patients with other oral anatomic subsites.

Oral squamous cell carcinoma (OSCC) classification is based on the Tumor, Lymph node, Metastasis (TNM) stage system (Supplemental Table 1, Supplemental Digital Content 1, http://links.lww.com/SCS/J468).

Modifications reported in the eighth Edition of the American Joint Committee on Cancer (AJCC) Staging Manual, published in 2017,^15^ include the addition of new variables to improve diagnostic accuracy. Among these new parameters is the “Depth of Invasion” (DOI), added to the “Tumor” category. DOI is defined as the distance from the basal membrane to the deepest tumor invasion point.^15^ DOI is considered a relevant histologic risk factor in oral carcinomas. It was found that the deeper a tumor is, the more likely it is that the disease will spread to the lymph nodes in the neck region.^16^

In addition to the DOI, the “Lymph node” category for extranodal extension has been implemented. Lastly, a further variation from the previous classifications involves a new and distinct staging system for HPV-positive patients.^17^

The treatment for OTSCC is strictly related to the tumor stage at diagnosis. For OTSCC in early stages (T1 or T2, N0, and low depth of invasion), wide local surgical excision (WLE) with free margins of 1 cm in width and depth,^18^ remains the mainstream treatment.^19^

The extent of the excision and the choice of the surgical technique, which may be more or less invasive, are strictly related to the cancer size and stage.^12^

The extent of surgical resection of tongue tissue (glossectomy) for tumor removal was first classified by Ansarin et al.^20^ The proposed classification, aimed at standardizing the surgical resection phase, identifies 5 types and 2 subtypes of glossectomy (Supplemental Table 2, Supplemental Digital Content 1, http://links.lww.com/SCS/J468).

Postoperative radiotherapy or chemotherapy should be indicated in cases of locally advanced OTSCC if specific factors such as perineural infiltration (PNI) or lymphovascular infiltration of the disease are present.^21^

The Oral tongue squamous cell carcinoma (OTSCC) is a type of cancer associated with a high rate of metastasis in the neck and an increased risk for skip metastasis. In cases of lymph node involvement, the treatment of choice is selective neck dissection with resection of the lymph nodes at highest risk of metastasis (levels I–IV).^22^

The following paper reports the results concerning 5 patients with early-stage oral tongue cancer treated with an innovative reconstructive surgical technique under local anesthesia. The proposed technique involves the removal of pathologic tissue via laser surgery and the reconstruction of the surgical wound, combining 2 biomaterials: cross-linked hyaluronic acid and a highly biocompatible, resorbable porcine pericardial membrane.

## METHODS

Clinical evaluations reported in this paper were conducted in accordance with the principles of the Declaration of Helsinki and the guidelines of the Azienda Ospedaliero-Universitaria of Parma.

The Ethics Committee (Area Vasta Emilia Nord AVEN Parma) approved the collection and sharing of data related to all patients included in this case series (Protocol number: 523/2025/PARERE/AOUPR).

The case series included 5 patients treated for early-stage OTSCC. In all cases, the incisional biopsy of the tongue lesion revealed the presence of an in situ/microinvasive carcinoma of the tongue with negative lymph node and distant metastases. For all 5 patients, surgical excision of the pathologic tissue was indicated, without postoperative radiotherapy or chemotherapy.

Three of the 5 patients were male (60%), and the remaining 2 were female (20%), with an average age of 62.6 years (minimum age=40 and maximum age=85).

The absence of risk factors was declared by 60% (n=3) of all patients. In this regard, only 1 patient was referred to as a smoker (20%), while the remaining 1 case (20%) was a former-smoker (from 1 y from the date of their first visit to our operating unit).

All 5 patients showed leukoplasic lesions that were nonhomogeneous in color and thickness, measuring more than 2 cm. Two of the 5 patients described had an initial diagnosis (established by the histopathologic examination of biopsy samples) of in situ carcinoma (40%); 1 (20%) had a diagnosis of low-grade dysplasia, 1 of pseudoepitheliomatous hyperplasia (20%), and in only 1 case (20%), a diagnosis of verrucous carcinoma in lichenoid dysplasia was made.

Concerning the lesion localization, and therefore the site of intervention, all lesions (n=5) were located on the tongue. In particular, 3 lesions were placed on the tongue’s margin (60%), and in 2 cases (40%), on the tongue’s back.

Information is detailed in Supplemental Tables 3 and 4, Supplemental Digital Content 1, http://links.lww.com/SCS/J468.

The outcomes analyzed included wound-healing times, postoperative symptoms reported by patients, any functional impairments, and functional alterations. The assessments were conducted 1 week, 3 weeks, and 1 month after surgery.

In all cases, the surgical removal of the lesion was performed with safety margins, extending into healthy tissue by 1 cm in width and depth. Excision was performed using a Neodymium Doped Yttrium Aluminum Garnet Laser (Nd:YAG, 1064 nm, output power 3.5 W; frequency: 60 Hz; fiber diameter: 300 μm). After bleeding control, a layer of cross-linked hyaluronic acid gel hyaDENT BG (Zurich, Switzerland: REGEDENT AG; composition: 1.6% cross-linked hyaluronic acid, 0.2% native HA) was applied (not infiltrated) to the bottom of the surgical defect, until complete coverage of the wound. To optimize the effect of cross-linked hyaluronic acid and to prolong the product’s permanence within the wound, the surgical site was finally covered with a membrane. In particular, in 1 case, a biocompatible, porcine pericardium resorbable membrane SMARTBRANE (Zurich, Switzerland: REGEDENT AG; composition: native porcine pericardium) and in 4 of the 5, a resorbable hemostatic agent made from oxidized and regenerated cellulose (SURGICEL) was applied over the cross-linked hyaluronic acid gel layer.

### Clinical Case Description

The following is a case of an 84-year-old female patient who presented at our operative unit with a white lesion on the right side of her tongue, measuring ∼5.0 × 4.0 cm. The patient reported a medical history of arterial hypertension, hypercholesterolemia, and hypothyroidism. On clinical inspection, the lesion appeared nonhomogeneous and irregularly bordered (Fig. 1); on palpation, it felt hardened and painful.

**FIGURE 1:**
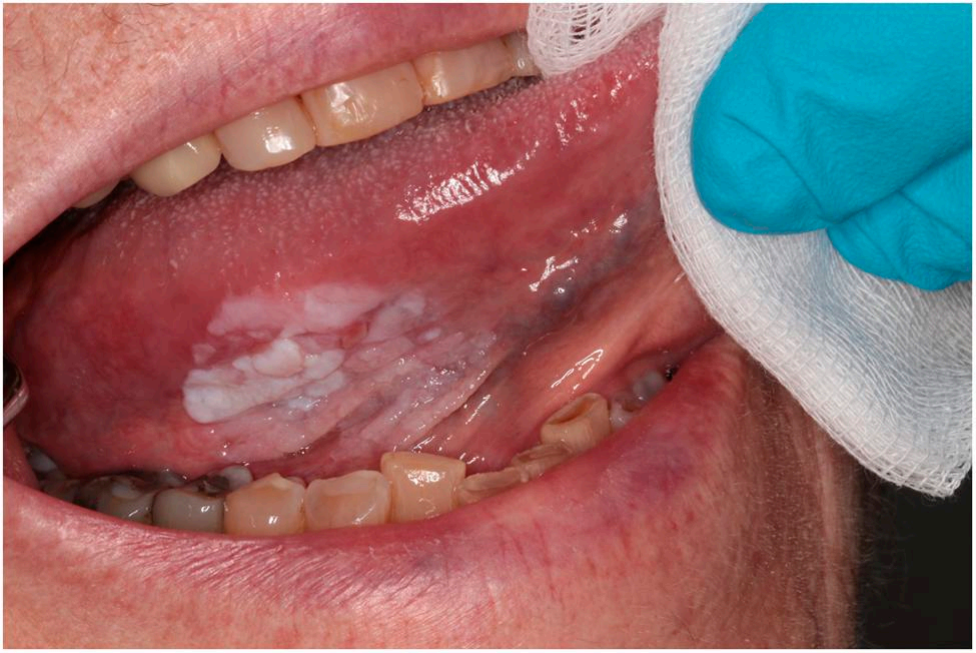
Clinical appearance of the lesion on first examination.

The incisional biopsy of the lesion revealed the presence of a hyperkeratosis and chronic lichenoid inflammation in low-grade dysplasia. Given the heterogeneous clinical appearance, the highly suspicious anatomic location, and the presence of dysplasia, it was decided to perform surgical removal of the lesion within safe margins.

The resection margins were delineated, extending at least 5 mm into healthy tissue at the periphery and deep to the lesion (Fig. 2A).^19^ The excision was performed using a Neodymium Doped Yttrium Aluminum Garnet (Nd:YAG) laser (1064 nm Fidelis Plus, Fotona, Ljubjana, Slovenia, 3.5 W, frequency: 60 Hz; fiber diameter: 300 μm, power density: 488.281 W/cm^2^) (Fig. 2B). Once the tissue to be excised was entirely removed (Fig. 2C), a layer of cross-linked hyaluronic acid gel was applied to the bottom of the surgical wound (Fig. 2D). Lastly a porcine pericardium resorbable membrane shaped according to the contour of the defect and hydratated with further xHyA (Fig. 2E), was placed on the wound and secured with resorbable sutures (Vicryl 5/0) (Fig. 2F).

**FIGURE 2:**
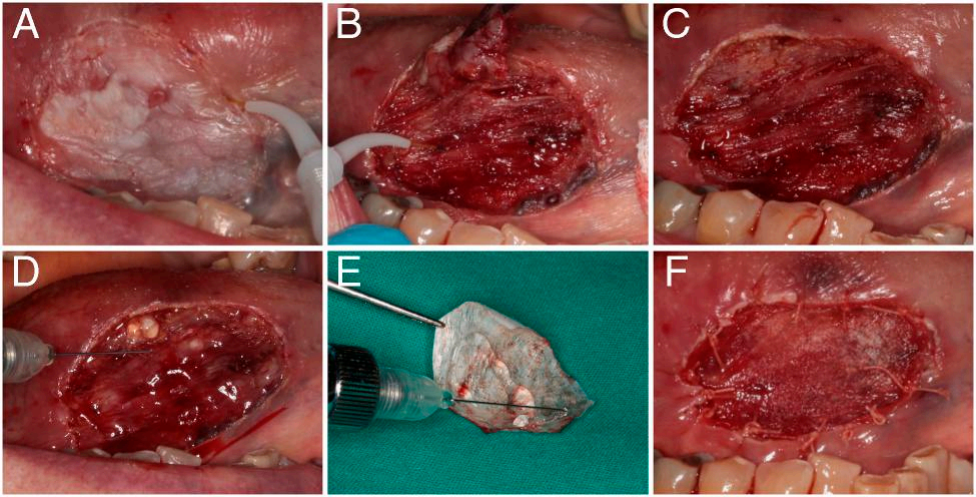
(A), (B), (C), (D), (E), and (F) are surgical phases.

The surgical piece (Fig. 3A-B) histopathologic examination revealed a well-differentiated, minimally invasive epidermoid carcinoma in lichen dysplasia and ulcerative area.

**FIGURE 3:**
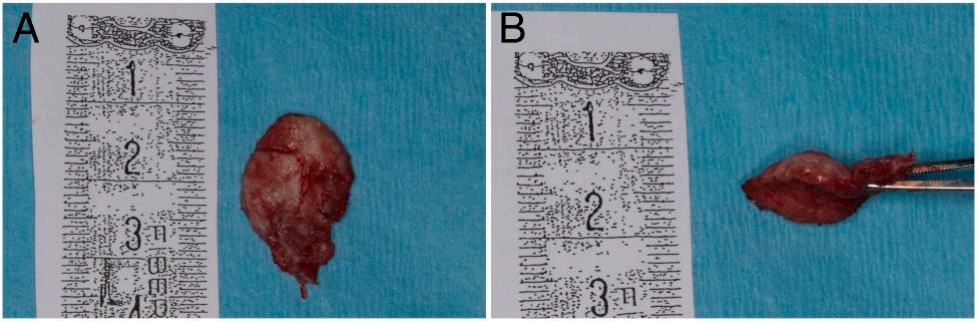
(A) and (B) is a surgical piece detail.

Per standard clinical practice, the patient underwent an MRI of the neck, which showed no lymph node involvement.

One week after surgical excision, the surgical site was entirely covered with fibrin, with no signs of inflammation (Fig. 4A).

**FIGURE 4:**
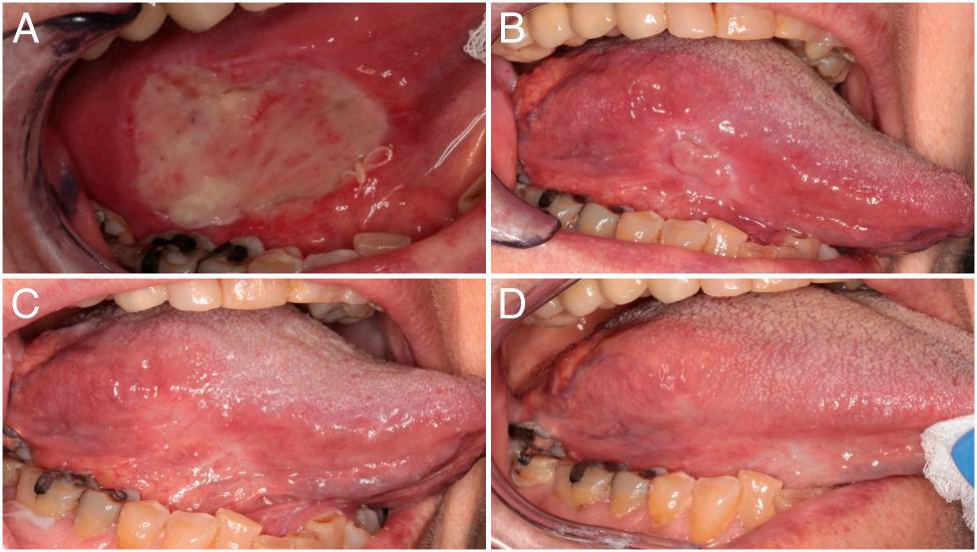
(A) One-week follow-up. (B) Three-week follow-up. (C) Two-month follow-up. (D) One-year follow-up.

During the 1-week follow-up, no pain or functional impairments were reported.

Three weeks after surgery, almost complete re-epithelization of the surgical wound was observed (Fig. 4B), and definitive re-epithelization was achieved within 2 months of surgery (Fig. 4C). One month after surgery, MRI scans with and without contrast were performed, confirming the absence of lymph node involvement. During the first year of follow-up, 2 neck ultrasounds were performed, and a second MRI scan 1 year later confirmed the absence of recurrence and lymph node involvement.

One year after surgery, no scarring at the surgical site was observed, and no functional alterations were recorded (Fig. 4D). Lastly, no signs of disease recurrence were detected.

## RESULTS

Assessments of the surgical wound appearance and healing quality, any symptoms reported by patients, any functional impairment, and complications were conducted at 1 week, 3 weeks, and 3 months after surgery.

In all treated cases (100%), after 1 week of intervention, the surgical wound was completely covered by fibrin, and no signs of inflammation were observed. Furthermore, none of the patients reported any functional impairment.

At the 3-week follow-up, the aforementioned assessments were repeated, with additional investigation for any postoperative complications. No differences from results recorded during the first follow-up were found, and no complications were detected.

## DISCUSSION

Surgical excision remains the gold standard treatment for both early and advanced stages of OSCC,^23^,^24^ with or without the addition of radiotherapy, chemotherapy, or neck lymph node dissection, depending on the stage of the cancer. Based on the extent of the cancer, tissue resection may result in more or less noticeable cosmetic defects and/or functional limitations (eg, difficulty speaking, eating, and opening the mouth).

In this context, reconstructive surgical techniques are pivotal, as they minimize cosmetic and functional complications by replacing the removed tissue.^25^ This has decidedly positive effects on patient acceptance of the therapy and allows for reaching aesthetic and physical trade-offs.^25^

The literature suggests that locoregional flaps, such as myomucosal local flaps in all currently available variants, Facial Artery Myomucosal flap (FAMM),^26^ FAMM reverse, Bozola et al,^27^,^28^ Zhao et al,^29^ and submental island flap^23^,^24^ are among the reconstructive surgical techniques most indicated for small and medium-sized defects of the oral cavity.^30^ More in detail, myomucosal flaps are associated with a tendency of oral mucosa cells to undergo faster healing with less risk of scarring,^31^ unlike free flaps (ie, the radial forearm free flap, RFFF, the traditional and modified lateral arm free flap, LAFF, mLAFF, and the anterolateral thigh flap, ALTF).^32^ The development of different variants of this surgical technique was designed to overcome the contraindication for each procedure and allow its applicability in various conditions.^33^ These flaps offer several advantages since they allow the replacement of the removed pathologic mucosal tissue with a tissue with similar characteristics,^34^ eliminate the need for an external visible incision, and minimize the risk of morbidity at the donor site, guaranteeing aesthetic and functional results.^31^,^35^,^36^ Furthermore, the use of myomucosal flaps maintains mucosal secretion and sensitivity, a requirement for more rapid and optimal recovery of tongue function in subsequent stages of rehabilitation.^28^,^34^

To date, the Bozola flap remains the best reconstructive option for T1, T2, and T3 OTSCC cases requiring type I, II, and IIIa glossectomy defects (according to Ansarin’s classification).^28^ As described in detail in a technical note by Salzano et al^28^ published in 2025, the Bozola flap has several advantages: first, it is the only reconstructive option among myomucosal flaps in cases of lymph node metastasis; secondly, the flap is highly elastic and malleable, characteristics that make the surgical procedure less complex and articulated in its phases. Finally, thanks to its range of rotation, it is easy to reach any site in the oral cavity, making the procedure possible regardless of the defect's location.

Although the aforementioned advantages are well documented in the literature, local flaps require the patient to undergo general anesthesia and a variable length of hospital stay. In addition to this, several complications seem to be related to these procedures, such as donor site necrosis, trismus, paralysis of the branch of the facial nerve, and incompetence of the labial commissure and retraction or fibrosis formation during the donor site healing process.^28^,^33^

With regard to the Bozola flap, the presence of the vascular pedicle, which inevitably crosses the masticatory line of the dental arches, exposes it to a risk of trauma. To minimize the likelihood of this occurring, in some cases the extraction of third molars is required, and in cases of reconstruction of more anterior areas of the tongue, the extraction of second molars may also be indicated.^35^

To address these limitations, synthetic, biocompatible grafts are promising options. The use of a surgical technique involving biomaterials eliminates the risk of secondary morbidity at the donor site, as it does not require graft harvesting from the patient. Furthermore, unlike secondary healing, it guarantees aesthetic and functional preservation of the intraoral and extraoral areas.

The present paper reports the qualitative results on postoperative outcomes of an innovative reconstructive surgical technique that combines the positive effects of cross-linked hyaluronic acid on tissue repair with the application of a biocompatible barrier.

In all cases described, the glossectomy was performed entirely with a Neodymium-Doped Yttrium Aluminum Garnet (Nd:YAG) laser (1064 nm). It is important to emphasize that the Nd:YAG laser has a strong affinity for hemoglobin and melanin, achieving complete hemostasis. The carbonization wave reaches ∼500 microns, ensuring tissue destruction that extends into healthy tissue for at least 2 mm, providing additional safety in the surgical margin.

Hyaluronic acid (HA) is a ubiquitous glycosaminoglycan naturally present in our body. In fact, in addition to being a main constituent of the extracellular matrix, this molecule is found in significant concentrations in epithelial, connective, and neural tissues, as well as in the vitreous humor and synovial fluid, where it acts as a lubricant and shock absorber.^37^,^38^ Hyaluronic acid in its native form is a molecule involved in several essential biological processes, thanks to its unique ability to bind to a wide range of molecules, including proteins, lipids, and carbohydrates.^38^ The limitation of the clinical use of native hyaluronic acid is its rapid biodegradation by hyaluronidases, with limited action over time. For this reason, a synthetic form of hyaluronic acid, cross-linked hyaluronic acid (xHyA), has been developed to overcome these clinical limitations. As described by Choi et al,^22^ a synthetic form is achieved through the use of specific reagents that induce the formation of cross-linked intermolecular and intramolecular bonds between different HA polymers.^39^ This structure confers higher resistance to mechanical stress and slower degradation compared with the native form.^29^ The clinical use of xHyA guarantees several advantages, such as the maintenance of postoperative functional integrity of the treated anatomic structure, reduction of postoperative pain,^40^ acceleration of the healing process,^41^ promotion of neoangiogenesis,^42^ bacteriostatic action, and lower risk of infection.^43^

In a recent paper, the Authors reviewed the literature on the use of cross-linked hyaluronic acid in oral soft tissue regeneration.^44^ Although there are several studies investigating the efficacy of this molecule in bone and periodontal tissue regeneration, there is a lack of studies evaluating the effect of xHyA on the oral mucosa.^44^

In the proposed technique, 2 types of membranes were used to isolate the surgical site from the oral environment: a porcine pericardium resorbable membrane and an oxidized regenerated cellulose gauze.

The porcine pericardium resorbable membrane is a strongly biocompatible biomaterial,^45^ thanks to meticulous cleaning processes using Supercritical carbon dioxide (scCO_2_), carried out during production.^46^ Moreover, this membrane promotes blood coagulation and allows cell adhesion, thus improving the natural regenerative process.^47^

SURGICEL is a homeostatic gauze, composed of oxidized regenerated cellulose (ORC), for example, a polyanion, whose functional unit is polyanhydroglucuronic acid. In surgery, ORC yields a safe device for managing mild to moderate bleeding, as it acts as a matrix for clot formation and promotes platelet activation.^39^,^48^ In addition to the hemostatic effect, its bactericidal and antibacterial action is also thoroughly described.^48^,^49^

Qualitative results from cases treated to date using the proposed surgical technique suggest the procedure's validity. Using a surgical reconstruction technique without a donor site would get rid of the risk of morbidity from the site and reduce postoperative discomfort. Furthermore, all cases mentioned were performed under local anesthesia, allowing the patient to avoid prolonged hospitalization. Finally, a significant advantage is the quality of healing achieved: while traditional reconstructive surgical techniques involve replacing the removed tissue with another (similar or significantly different), in the instance of the proposed technique, the placement of the resorbable membrane in the porcine pericardium allows anatomic continuity to be recreated in the context of the wound, acting as a scaffold for the repair cells, thus allowing the formation of tissue identical to that removed.

The cases reported in the article would seem to support the advantages of the reconstructive technique using hyaluronic acid, as they are all associated with faster wound healing and a highly favorable postoperative course. Indeed, based on our experience, qualitative assessments of healing time, postoperative symptoms, functional impairment, and complications corroborate the claim that cross-linked hyaluronic acid promotes faster healing of the surgical site and a favorable postoperative course, with no functional limitations or pain following the procedure.

Despite this, it should be noted that to validate the proposed surgical technique, a randomized controlled single- or double-blind study would be necessary, and the results obtained would then be compared with those achievable with traditional reconstructive surgical techniques. Ultimately, the positive results reached from our observations warrant further investigation through clinical trials to assess their efficacy and safety.

## Supplementary Material
