# Supplementary material for: Reconstruction of Surgical Defects From Early Carcinoma of Tongue Supported by Cross-Linked Hyaluronic Acid
Source: J Craniofac Surg. 2026 May 5;37(11-12):3151–6. doi: 10.1097/SCS.0000000000012815 (PMC13764228; doi:10.1097/SCS.0000000000012815)
Supplement: Supplementary file 1 [file scs-37-03151-s001.pdf]

Table 1 Oral Cancer TNM Classification - 8th Edition

**TUMOR STAGING (T)**		**CLINICAL LYMPH NODE STAGING (cN)**		**PATHOLOGICAL LYMPH NODE STAGING (pN)**		**DISTANT METASTASIS (M)**	
Tis	Carcinoma in situ	cN0	No regional lymph node metastasis	pN0	No regional lymph node metastasis	cM0	No distant metastasis
T1	Tumor ≤2 cm with DOI ≤5 mm	cN1	Metastasis in single ipsilateral lymph node metastasis ≤3 cm in greatest dimension and ENE(−)	pN1	Metastasis in a single ipsilateral lymph node metastasis ≤3 cm in greatest dimension and ENE(−)	cM1	Distant metastasis
T2	Tumor ≤2 cm and DOI >5 mm<br>*or* tumor >2 cm and ≤4 cm with DOI ≤10 mm	cN2a	Metastasis in a single ipsilateral lymph node >3 cm and <6 cm and ENE(−)	pN2a	Metastasis in single ipsilateral lymph node >3 cm and ≤6 cm and ENE(−)<br>*or* single ipsilateral lymph node ≤3 cm and ENE(+)	pM1	Distant metastasis, microscopically confirmed
T3	Tumor >2 cm and ≤4 cm with DOI >10 mm<br>*or* tumor >4 cm with DOI ≤10 mm	cN2b	Metastases in multiple ipsilateral lymph nodes, none larger than 6 cm and all ENE(−)	pN2b	Metastases in multiple ipsilateral lymph nodes, none larger than 6 cm and all ENE(−)		
T4a	Tumor >4 cm with DOI >10 mm<br>*or* tumor invades adjacent structures only (e.g., through cortical bone of the maxilla or mandible or involves maxillary sinus, skin of the face	cN2c	Metastasis in bilateral or contralateral lymph nodes, none larger than 6 cm and all ENE(−)	pN2c	Metastases in bilateral or contralateral lymph nodes, none larger than 6 cm and all ENE(−)		
T4b	Tumor invades masticator space, pterygoid plates, skull base, and/or encases internal carotid artery	cN3a	Metastasis in a lymph node larger than 6 cm and ENE(−)	pN3a	Metastasis in any lymph node larger than 6 cm and ENE(−)		
		cN3b	Metastasis in any lymph node with clinically overt ENE(+)	pN3b	Metastasis in any lymph node >3 cm and ENE(+),<br>*or* multiple ipsilateral lymph nodes of any size with ENE(+),<br>*or* any number of contralateral lymph nodes ENE(+)		

Table 2 Glossectomy classification. Ansarin et al. 2019

**TYPE I**<br>*(mucosectomy)*	Involves the removal of the mucosa and submucosa with an extension into healthy tissue of 0.5–1.0 cm (depending on the type of lesion). Regarding the deeper portion, it is recommended to include a thin layer of the intrinsic muscles.	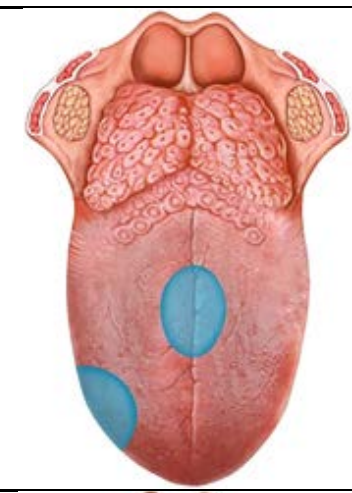 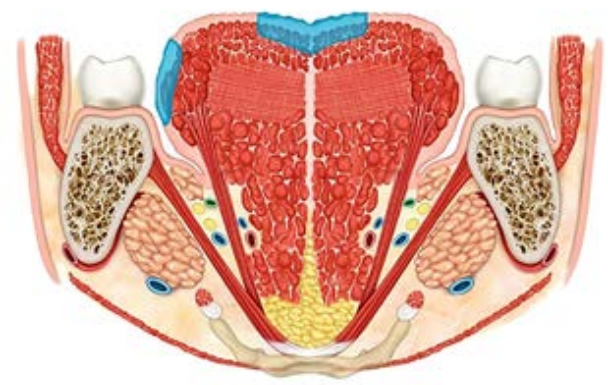
**TYPE II**<br>*(partial glossectomy)*	Involves the removal of the mucosa, submucosa and intrinsic muscles, down to the surface of the extrinsic muscles. In this second case, the margin of healthy tissue reaches 1.5 cm in all directions. The terminal branches of the lingual artery must be ligated, while the lingual nerve is preserved.	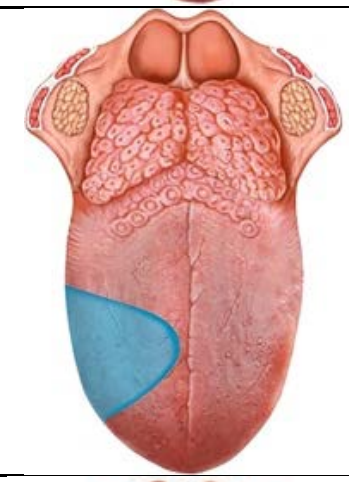 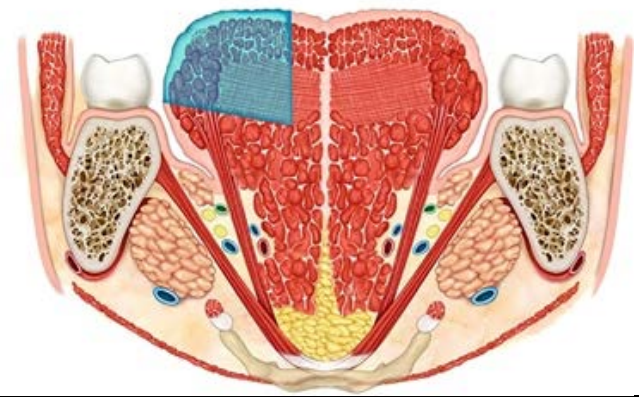
**TYPE IIIa**<br>*(hemiglossectomy)*	Involves the removal of the mucosa, submucosa, and intrinsic and extrinsic muscles ipsilateral to the lesion. Once again, it is essential to maintain safety margins of at least 1.5 cm. This type of glossectomy also involves the *en bloc* removal of the lingual artery, lingual nerve, and hypoglossal nerve. The base of the ipsilateral tongue is preserved.	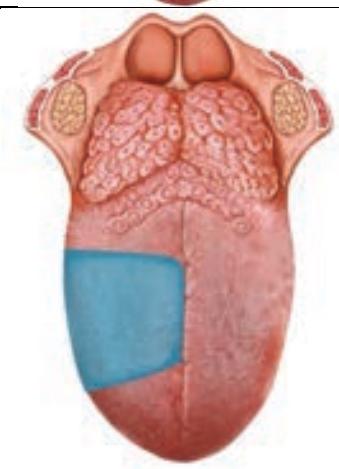 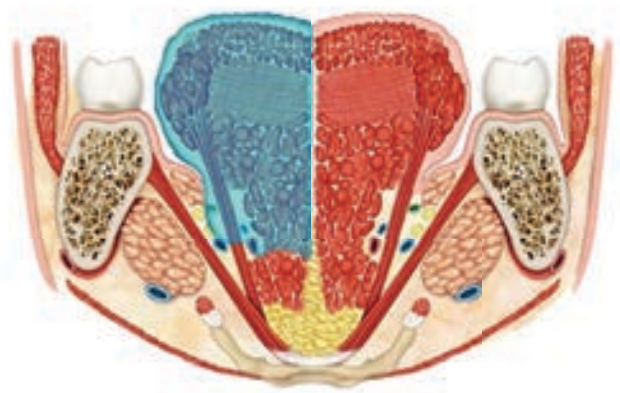
**TYPE IIIb**<br>*(compartimental hemiglossectomy)*	Involves the removal of the mucosa, submucosa, intrinsic and extrinsic muscles ipsilateral to the lesion, genioglossus, hyoglossus and styloglossus muscles, and the inferior portion of the palatoglossus muscle. The midline raphe in included in the resection. The lingual nerve is resected as far cranially as possible. The hypoglossal nerve is removed after the ansa, the lingual artery and vein is ligated in proximity to the horn of the hyoid bone, and removed en bloc with specimen and neck nodes.	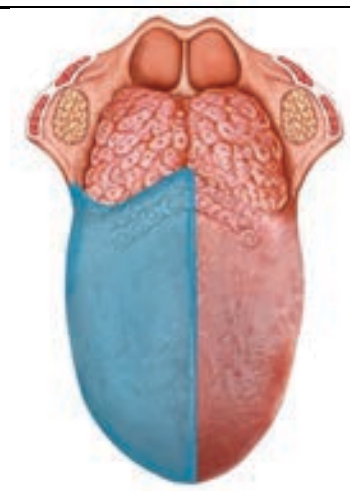 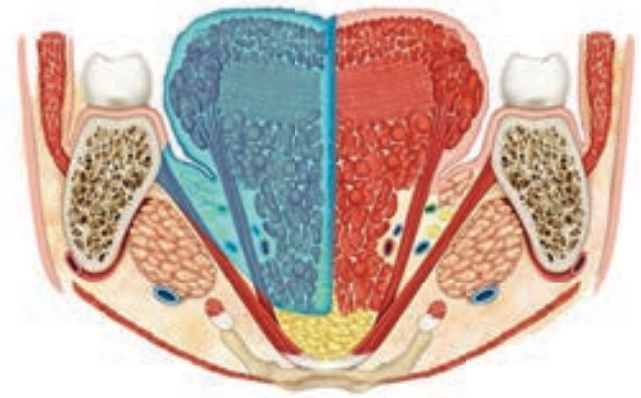
**TYPE IVa**<br>*(subtotal glossectomy)*	Involves the anterior subtotal glossectomy with maintenance of both sides of the base of the tongue. Hyoglossus muscle and hypoglossal and lingual nerves from the less involved side, are also preserved.	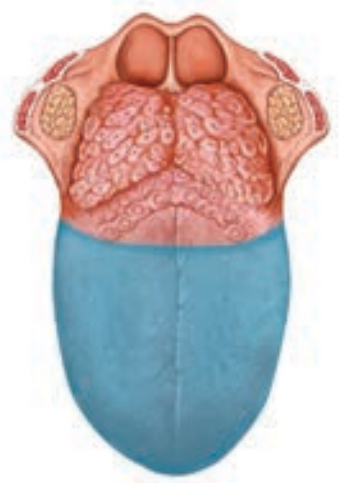 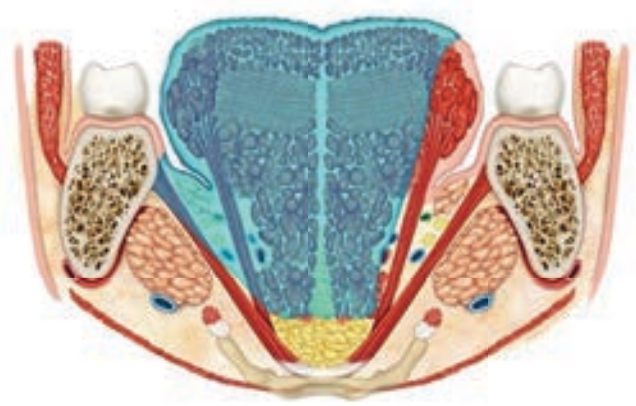

**TYPE IVb**<br>*(near-total glossectomy)*	Involves the anterior subtotal glossectomy with extension to the ipsilateral base of the tongue. Hyoglossus and styloglossus muscles, hypoglossal and lingual nerves, and lingual artery are preserved.	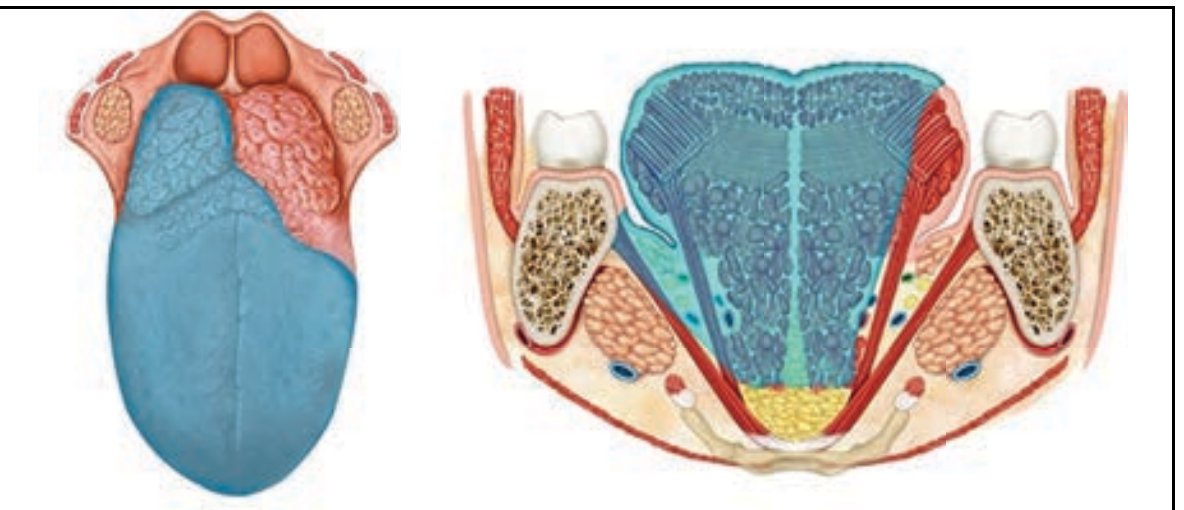
**TYPE V**<br>*(total glossectomy)*	Involves the complete removal of the mobile tongue: the base of the tongue is transected at the level of the vallecula.	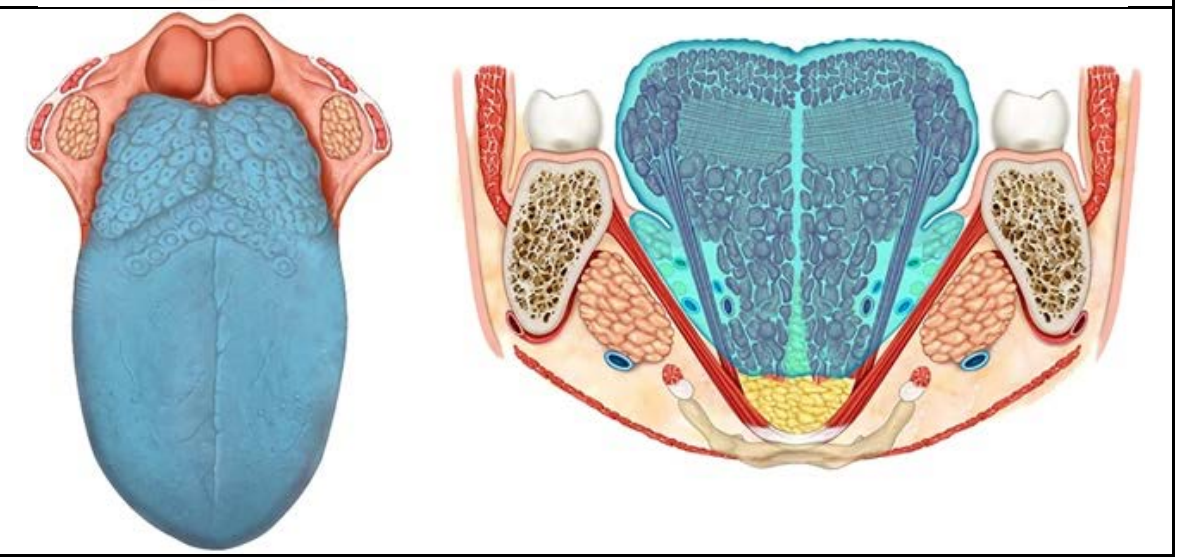

Table 3 Data of included patients

#	Gender/Age	Medical history	Therapies	Risk factors
1	F/85	Hypertension, hypercholesterolemia, hypothyroidism	Antihypertensives, statins, anxiolytics, eutirox	Former smoker
2	M/40	-	-	-
3	F/70	HCV positive	-	-
4	M/64	-	-	Former smoker (since 2023)
5	M/57	-	-	-

Table 4 Detail of treated lesions. The glossectomy extent was established based on the classification of Ansarin, published in 2019

#	Lesion localization	Pre-operative biopsy	Operative technique	Glossectomy classification [21]	Final diagnosis	TNM Stage
1	Right tongue margin	Mild epithelial hyperplasia with hyperkeratosis and chronic lichenoid inflammation. Low-grade dysplasia	Nd:YAG+xHyA+PPRM	II	Well differentiated, minimally invasive epidermoid carcinoma in lichenoid dysplasia	pT1<br>DOI<1mm
2	Left tongue margin	Focal area of severe epithelial dysplasia	Nd:YAG+xHyA+Surgicel TM	II	Well differentiated keratinizing epidermoid carcinoma, initially infiltrating	T1N0<br>DOI=1mm
3	Back of the tongue	Verrucous Carcinoma in lichenoid dysplasia	Nd:YAG+xHyA+Surgicel TM	II	Verrucous, well-differentiated carcinoma, initially infiltrating the lamina propria	T1N0<br>T<2mm
4	Back of the tongue	Pseudoepitheliomatous hyperplasia with hyperkeratosis	Nd:YAG+xHyA+Surgicel TM	II	Severe lichenoid dysplasia and hyperkeratosis	Tis
5	Right tongue margin	Well-differentiated verrucous carcinoma (in situ)	Nd:YAG+xHyA+Surgicel TM	II	Verrucous Carcinoma	Tis
